# Filling the Gaps in Antagonist CCR5 Binding, a Retrospective and Perspective Analysis

**DOI:** 10.3389/fimmu.2022.826418

**Published:** 2022-01-19

**Authors:** Yerkezhan Amerzhanova, Luca Vangelista

**Affiliations:** Department of Biomedical Sciences, School of Medicine, Nazarbayev University, Nur-Sultan, Kazakhstan

**Keywords:** CCR5, CCL5, antagonist, binding, rational design

## Abstract

The large number of pathologies that position CCR5 as a central molecular determinant substantiates the studies aimed at understanding receptor-ligand interactions, as well as the development of compounds that efficiently block this receptor. This perspective focuses on CCR5 antagonism as the preferred landscape for therapeutic intervention, thus the receptor active site occupancy by known antagonists of different origins is overviewed. CCL5 is a natural agonist ligand for CCR5 and an extensively studied scaffold for CCR5 antagonists production through chemokine N-terminus modification. A retrospective 3D modeling analysis on recently developed CCL5 mutants and their contribution to enhanced anti-HIV-1 activity is reported here. These results allow us to prospect the development of conceptually novel amino acid substitutions outside the CCL5 N-terminus hotspot. CCR5 interaction improvement in regions distal to the chemokine N-terminus, as well as the stabilization of the chemokine hydrophobic core are strategies that influence binding affinity and stability beyond the agonist/antagonist dualism. Furthermore, the development of allosteric antagonists topologically remote from the orthosteric site (e.g., intracellular or membrane-embedded) is an intriguing new avenue in GPCR druggability and thus a conceivable novel direction for CCR5 blockade. Ultimately, the three-dimensional structure elucidation of the interaction between various ligands and CCR5 helps illuminate the active site occupancy and mechanism of action.

## Introduction

CCR5 is a chemokine receptor belonging to the GPCR superfamily. CCL5 is a proinflammatory chemokine, largely studied for the pathophysiological implication of CCR5 engagement. The CCR5:CCL5 axis proved to be a crucial crossroad for a large number of infections and inflammatory conditions (including HIV-1 and *Staphylococcus aureus* infections, cancer and atherosclerosis). In most of these pathologies, CCR5 blockade is a promising therapeutic avenue (1, 2). CCR5 antagonist occupancy of the receptor active site has a dual advantage: it stabilizes the receptor in an inactive conformation, and it allows competitive binding with the natural agonist ligands (e.g., CCL3, CCL4 and CCL5) or the microorganism ligands (e.g., HIV-1 gp120 and *S. aureus* LukED toxin). Interestingly, both gp120 and LukED pathogenic engagement of CCR5 has been reported not to activate the receptor (3, 4). However, the sequence variability presented by the diversity of HIV-1 strains is reflected in the molecular determinant for CCR5 binding (i.e., the V3 loop), possibly generating an array of binding modes (including agonist binding) and CCR5 conformation occupancy (5). CCR5 antagonism is a mandatory pharmacological intervention for inflammatory conditions caused by receptor activation and a rational approach to combat HIV-1 infection. The availability of maraviroc (MVC), a small chemical compound approved as drug for HIV-1 entry inhibition, allows the prompt investigation of CCR5 biochemical blockade beyond HIV-1 infection (6–8). Pertaining to HIV-1 inhibition, a large body of research has been focusing on the development of potent N-terminally-modified chemokine ligands of CCR5, mostly based on CCL5. Initially, CCL5 variants retained CCR5 agonist activity, later followed by efforts to attain a switch to antagonism (9). Last generation CCL5 derivatives acting as CCR5 antagonists could be a valid alternative to MVC, as they present *in vitro* anti-HIV-1 activity largely superior to MVC (10, 11). Monoclonal antibodies (mAbs) acting as CCR5 antagonists have been the subject of intense investigation (12) leading to promising therapeutic perspectives (13, 14). However, to date there is no information on the structural details of the interaction between these mAbs and CCR5. Hence, although extremely interesting, the structural understanding of their CCR5 blockade is to date relatively limited. Rational drug design approaches show that MVC is also prone to molecular improvements (15). The dualism on small chemical compound *versus* protein based CCR5 antagonists is *de facto* a territory in which information crosstalk may boost the advancement of both molecular classes.

In this study, we investigated both retroactively and in perspective the innovative role of selected point mutations inserted in CCL5 regions distal to the classically targeted N-terminus (11). We also briefly discuss emerging allosteric strategies to block GPCRs alternative to the active site occupancy and that could be of interest to tackle CCR5 in pathology.

## Methods

### Modeling Full-Length CCR5 in Complex With CCL5 5P12 5M and Retrospective Analysis

The high-resolution structure of CCR5 in complex with 5P7 CCL5 (a CCR5 antagonist) (PDB ID: 5UIW) (16) was used as template for the modeling of CCL5 5P12 5M, the most potent CCR5 antagonist reported to date (11). Six separate 3D models (T7L, F12Y, A13V, Y27W, F28W and E66S), accounting for the differences between 5P7 CCL5 and CCL5 5P12 5M were built using SWISS-MODEL. To compare the quality of the models, we used I-TASSER, Phyre2 and ModWeb. The model of CCL5 5P12 5M built on the 5UIW coordinates in all the platforms was used as a prototype to validate the most reliable superposition with the 5P7 CCL5 structure, and we decided that SWISS-MODEL provided the best output for the subsequent modeling and analysis.

Next, we modeled on 5UIW the first 15 AA of CCR5 N-terminus from the NMR solution structure of monomeric CCL5 bound to a synthetic doubly sulfated CCR5 N-terminus peptide (PDB ID: 6FGP) (17). We used homology modeling and data-driven flexible refinement to generate the final full-length model.

A retrospective structure-guided analysis was conducted to examine the quality and effect the six AA differences between 5P7 CCL5 and CCL 5P12 5M exerted on the full CCR5 model. Structures were visualized and analyzed using the PyMOL software.

## Results

### CCR5 Antagonist Active Site Occupancy and Its Role in Pathology

**Figure 1:**
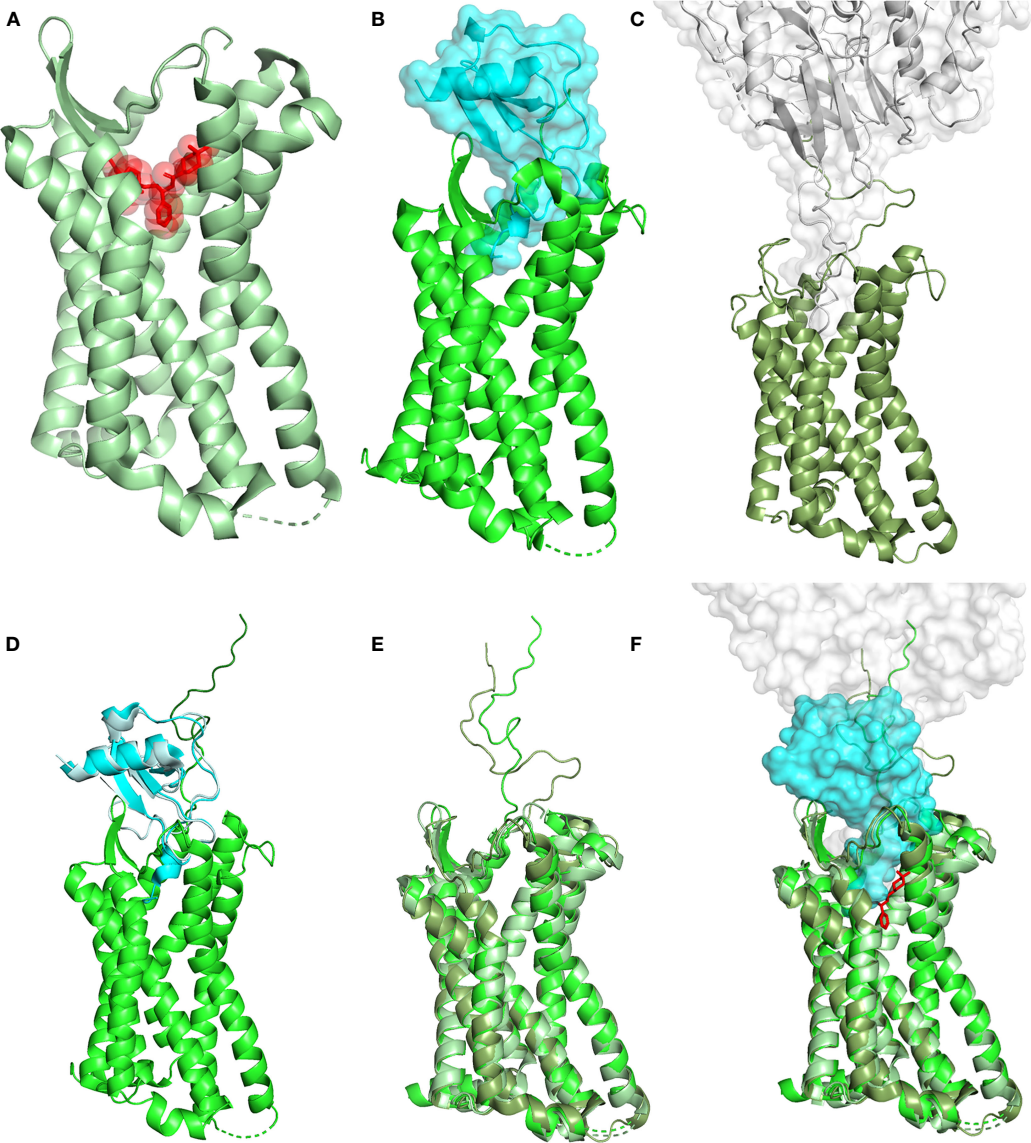
Structural landscape of antagonist CCR5 occupancy. **(A)** Crystal structure of the CCR5 (ribbon, pale green) in complex with the HIV-1 entry inhibitor MVC (transparent surface and sticks, red) (PDB ID: 4MBS). **(B)** Crystal structure of CCR5 (ribbon, green) in complex with high potency HIV-1 entry inhibitor 5P7 CCL5 (transparent surface and ribbon, cyan) (PDB ID: 5UIW). **(C)** Cryo-EM structure of a full-length gp120 (transparent surface and ribbon, gray) in complex with unmodified human CCR5 (ribbon, smudge green) (PDB ID: 6MET). **(D)** Full-length CCR5 (green) (from 5UIW) with the N-terminus (dark green) modeled upon fusion of the first 15 AA from the N-terminal segment of CCR5 in complex with wild-type CCL5 (pale cyan) (PDB ID: 6FGP). CCL5 (from 6FGP) and 5P7 CCL5 (cyan) (from 5UIW) were superimposed to allow reliable modeling. **(E)** Superimposition of CCR5 (ribbon) from 4MBS, 6MET and the modeled full length (5UIW/6FGP); color code as in **(A–C)**. **(F)** Superimposition of **(A, B)**, using the full length CCR5 model in **(D, C)**. CCR5 (ribbon), MVC (sticks), 5P7 CCL5 and gp120 (transparent surface). Color code as in **(A–C)**. Structural representations were generated using PyMOL.

CCR5 stabilization in an inactive conformation by the use of compounds capable to occupy the active site as antagonists may play a significant therapeutic role in several pathologies (**Figure 1**). Ample occupancy of the active site might not seem to be a necessary requirement in inflammatory conditions involving CCR5, however prevention of receptor engagement by natural chemokine agonists (e.g., CCL3, CCL4 and CCL5) needs to be attained to warrant full therapeutic efficacy. The differential efficiency by which MVC and CCL5 derivatives compete with natural chemokines needs to be investigated in detail. Receptor active site full occupancy becomes crucial in HIV-1 entry inhibition as this prevents the insurgence of resistant strains. The deep but limited CCR5 active site area occupied by MVC (**Figure 1A**) (18), as compared to the large surface of interaction covered by gp120 (**Figure 1C**) (3), allows HIV-1 to eventually raise MVC-resistant strains. Conversely, full occupancy of CCR5 active site by CCL5 derivatives (**Figure 1B**) (16) should prevent the emergence of HIV-1 resistant strains (19, 20). The different extent of the CCR5 interaction by MVC, gp120 and the CCL5 variant 5P7 CCL5 (a CCR5 antagonist) is highlighted in **Figure 1F**. It can be appreciated how the presence of 5P7 CCL5 would not allow any penetration of the receptor by gp120, nor any obvious molecular rearrangement leading to HIV-1 resistance could be envisaged. Interestingly, the 3D structure of CCR5 from the three complexes with antagonists is largely overlapping (**Figure 1E**), denoting similar inactive conformations.

### Improving CCR5 Engagement by Modifying CCL5 Distal to the N-Terminus

Among the CCL5 derivatives efficiently blocking CCR5, the most potent HIV-1 entry inhibitors reported to date are CCL5 5P12 5M and CCL5 6P4 5M, a CCR5 antagonist and a superagonist, respectively (11). Five mutated hotspots (5M) were selected to enhance different features related to the CCL5:CCR5 interaction. The classic E66S mutation is well known to promote disruption of CCL5 oligomerization (21, 22), a feature that favors an increase in CCR5 engagement by the availability of a larger number of CCL5 monomers in solution (11). F12Y and A13V mutations were introduced to eliminate a proteolytic process occurring during the production of the CCL5 variants in lactobacilli (23). These positions were also known to be crucial for CCR5 binding, particularly F12 (24, 25), yet both F12Y and A13V mutations led to an increase in anti-HIV-1 activity. Interestingly, the 12-14 AA stretch has been shown to be involved in CCL5 dimerization (22), and the F12Y and A13V might have influenced this feature, although this aspect has not been investigated and the 5P12 N-terminus leads to a monomeric CCL5 derivative (26). The Y27W and F28W mutations were introduced following consistent evidence of their improvement in anti-HIV-1 activity on small CCL5 peptide derivatives (27). The aggregated five mutations generated CCL5 5M, a CCR5 agonist with anti-HIV-1 activity comparable to 5P12 CCL5 and 6P4 CCL5 (11). Finally, the natural N-terminus of CCL5 5M was replaced with the 5P12 and 6P4 amino acid stretches (10), obtaining the CCR5 antagonist CCL5 5P12 5M and the superagonist CCL5 6P4 5M (11).

### Retrospective Analysis of CCL5 5P12 5M Interaction With CCR5

**Figure 2:**
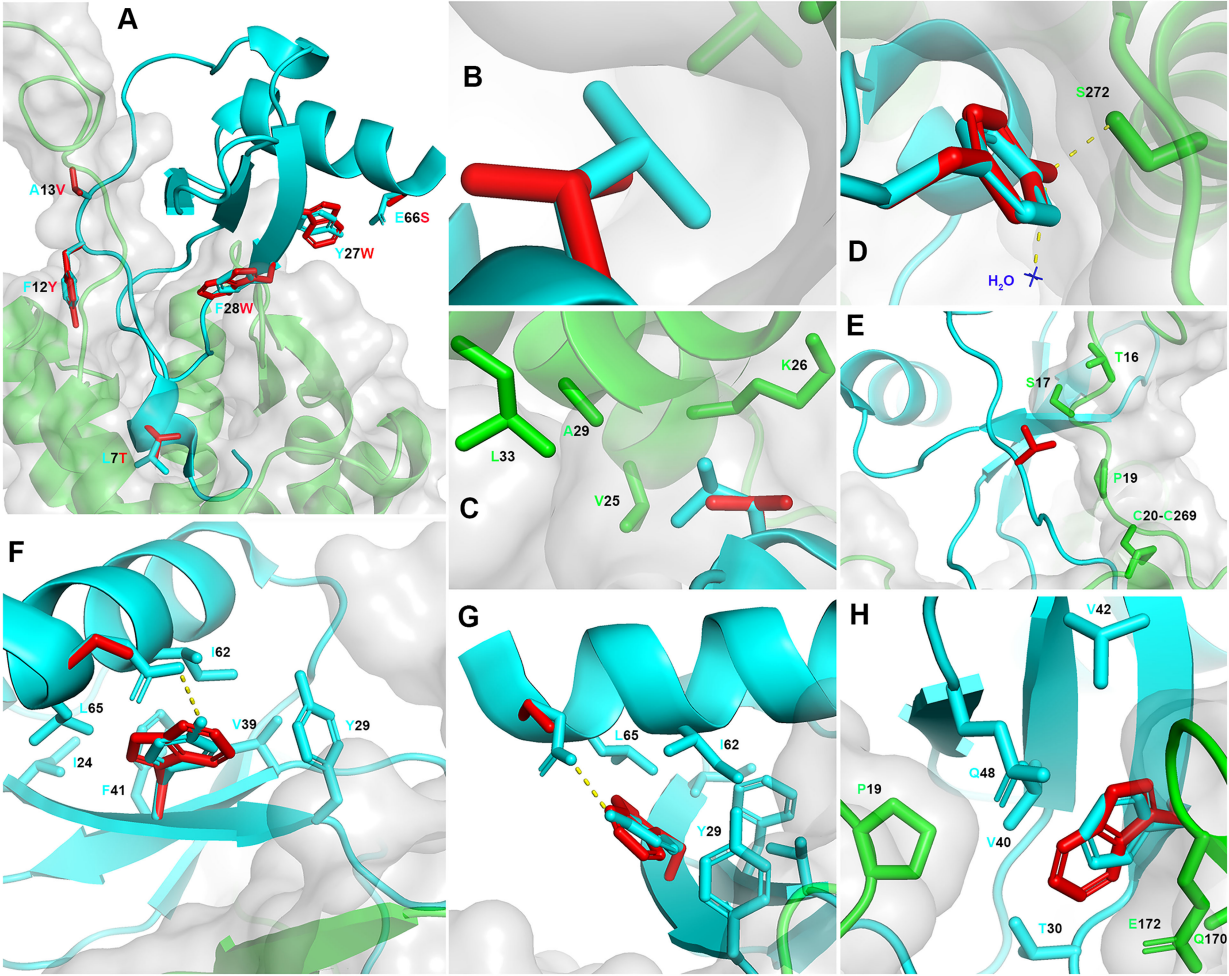
Retrospective structural analysis of CCL5 5P12 5M. **(A)** Overview on CCL5 5P12 5M modeled on 5P7 CCL5 (ribbon, cyan) and complexed with the modeled full length CCR5 (grey transparent surface and ribbon, green). In red, the six CCL5 5P12 5M residue side chains (sticks) that differ from 5P7 CCL5 (original side chains in stick, cyan): L7T, F12Y, A13V, Y27W, F28W and E66S. **(B, C)** Higher occupancy by L7 (cyan) compared to T7 (red) and proximity of V25, K26, A29 and L33 (CCR5). **(D)** Similar occupancy by F12 (cyan) and Y12 (red), with two extra hydrogen bonds with one molecule of water (blue) and S272 (CCR5). **(E)** Larger hydrophobic volume by V13 (red) compared to A13 (cyan), packing to S17 (CCR5) and lock of the CRS1.5 site from the other side of P19 (CCR5). **(F, G)** Y27 and E66 (cyan) form an hydrogen bond, eliminated in W27 and S66 (red). Conversely, W27 occupies more space (partly provided by S66) and packs against Y29, I62 and L65 in the chemokine hydrophobic core and presents more distal packing with I24, V39 and F41, possibly stabilizing the protein fold. **(H)** Substitution of F28 (cyan) with W28 (red) appears to be conservative, yet with an enhancement of a dual role: increase surface of interaction with P19, Q170 and E172 (CCR5) and enhanced chemokine hydrophobic core packing by facing T30, V40, V42 and Q48. Structural representations were generated using PyMOL.

The CCL5 5M derivatives have been generated in the absence of the 3D structural details of the CCL5:CCR5 interaction interface, and were the result of previous findings derived from CCL5 short peptides with anti-HIV-1 activity (27). The crystal structure of CCR5 in complex with 5P7 CCL5 (16) was published after submission of the work on the CCL5 5M variants (11). We therefore propose here a retrospective analysis of CCL5 5P12 5M, modelled upon the available crystal structure of 5P7 CCL5:CCR5, and reveal the insights into the structural details of the previously reported mutations that led to the highly potent anti-HIV-1 activity (**Figure 2**). In order to fully analyze and understand the structural implications of the mutations introduced in CCL5 5P12 5M, we modeled the CCR5 N-terminus on the crystal structure of the complex with 5P7 CCL5. This was made possible by the availability of the NMR structure of the complex between CCL5 and the N-terminus of CCR5 in solution (17). By superimposing CCL5 from 6FGP on 5P7 CCL5 from 5UIW we could complete the missing portion of CCR5 N-terminus (**Figure 1D**). Of interest, Abayev et al. generated a full CCR5 model of the interaction with CCL5 (17).

The CCR5 antagonist 5P12 CCL5 has been selected as lead compound for drug development (28), and, as a follow up, 5P12 was the N-terminus adopted to convert CCL5 5M into an antagonist (11). However, among the CCL5 derivatives obtained in the phage display study (10), 5P7 CCL5 resulted as the most stable CCR5 ligand for crystallization (16). With the aim of inspecting the differences with 5P7 CCL5 in its interaction with CCR5, the 3D structural model of CCL5 5P12 5M was generated upon the 5UIW coordinates and complexed with the modeled full CCR5 (**Figure 2A**). The 5P7 and 5P12 N-termini differ from each other by a leucine or a threonine in position 7, respectively. Indeed, the CCR5 hydrophobic environment around CCL5 position 7 appears to be better filled by a leucine than a threonine (**Figures 2B, C**). In light of this evidence, we produced a 5P7 version of CCL5 5M (unpublished). F12 has long been considered a crucial residue for the interaction with CCR5 (24, 25), an evidence confirmed by the 3D structure of the 5P7 CCL5:CCR5 complex (16). Nevertheless, the analysis of the F12Y mutation reveals two possible supplementary hydrogen bonds made by the tyrosine hydroxyl group with CCR5 S272 and one water molecule present in the crystal of the complex (**Figure 2D**), likely accounting for the observed increase in anti-HIV-1 activity (11). Compared to wild type alanine, a valine in position 13 appears to pack better with CCR5 S17 and P19 (**Figure 2E**). Taking into consideration the conserved chemokine-receptor pattern of interaction (16), with three chemokine recognition sites (CRS1, CRS1.5 and CRS2), V13 forms a sort of lock from the other side of CRS1.5, defined by the packing of P19 (CCR5) with the chemokine disulfide bond (CCL5 C11-C50). The Y27W and E66S mutations appear to complement each other, with the elimination of the original hydrogen bond between E66 and Y27 replaced by a larger space available for the bulky W27 thanks to the short side chain of S66 (**Figures 2F, G**). Moreover, W27 packs very well within the chemokine hydrophobic core by contacting Y29, I62 and L65, and more distal with I24, V39 and F41. This should possibly stabilize the protein fold and provide an indirect contribution to CCR5 engagement. Interestingly, W27 as well as the original tyrosine do not interact with the receptor. Hence, both S66 and W27 promote higher anti-HIV-1 potency compared to their native counterparts (E66 and Y27) by indirect molecular mechanisms not involving CCR5 binding. Finally, the F28W mutation results in a higher occupancy of the CCR5 space as well as an enhanced hydrophobic core packing of the chemokine. W28 is surrounded by several AA on both sides: CCR5 provides P19, Q170 and the alkyl portion of E172 side chain, while the chemokine face presents T30, V40, V42 and Q48 (**Figure 2H**). The original F28 presents a similar packing, however the larger hydrophobic surface offered by a tryptophan residue justifies the observed increase in anti-HIV-1 potency (11), as this likely reflects a tighter binding to the receptor. Interestingly, CCR5 P19, a crucial component of CRS1.5 appears to be embraced by the longer hydrophobic arms of V13 and W28, as compared to the original A13 and F28. Mutations Y27W, F28W and E66S might also influence the capacity of CCL5 to oligomerize, particularly in the oligomeric form reported in Wang et al. (29). The bulky tryptophan residues lead to a most likely unfavorable condition for oligomerization, given the tight packing observed at the interface for oligomerization (PDB ID: 2L9H), where residues 27 and 28 interact very closely with each other both intramolecularly and intermolecularly (at the dimer of dimers interface).

Further inspection of the CCR5 binding cavity and the extensive occupancy by 5P7 CCL5 allowed the identification of at least two new positions amenable of amino acid substitution. These substitutions have been tested by the SWISS-MODEL generation of 3D models of the CCL5 mutants that indicated a likely further enhancement in receptor occupancy and therefore a possible increase in the potency of the resulting CCL5 mutants (unpublished).

The analysis presented here points to the possibility to improve CCL5 in its interaction with CCR5 by modifying residues distal to the chemokine N-terminus, either by direct receptor affinity increase or by stabilization of the chemokine hydrophobic core.

## Discussion and Perspectives

Two recent reports shed light on CCR5 activation, with the 3D structure determination of CCL5:CCR5 and CCL3:CCR5 complexes in presence of the G_i1_ protein, as well as a constitutively activated CCR5 coupled to G_i1_, and the 6P4 CCL5:CCR5 complex also in presence of G_i1_ (30, 31). Differences in CCR5 conformation between inactive and active states can now be appreciated, as well as the chemokine N-terminus determinants that lead to receptor activation, fully opening the area to rational drug design. It is assumed that the major determinant for the affinity towards chemokine receptors is provided by the core of the chemokine, while the N-terminus is responsible for receptor activation (30, 31), however this might not be entirely true for the highly modified N-terminus in CCL5 derivatives such as 5P7 and 6P4. Indeed, the agonist CCL5 5M derivative reached very potent anti-HIV-1 activity in absence of N-terminal modifications (11). An analysis similar to the one reported in **Figure 2** can be envisaged for the CCL5 6P4 5M derivative (11), with largely similar structural implications for the core mutations incorporated in CCL5 5M.

Once aiming at the use of CCL5 derivatives as therapeutics, an important aspect that needs to be considered is the possible loss of tolerance due to the insertion of mutated amino acids in the wild type chemokine. In certain therapeutic settings, this may result in limited efficacy due to elimination by antibodies directed against the modified chemokine. With the availability of the molecular details of the CCR5 complexes with 5P7 CCL5, CCL5 and 6P4 CCL5 (16, 30, 31), the development of a CCR5 antagonist based on a limited modification of CCL5 N-terminus might be conceivable. Complemented by sparse mutations in the chemokine core to improve receptor affinity, this might be a valid strategy to prevent loss of tolerance and create CCL5 variants that fall below the threshold of immune detection.

Changing perspective, away from the orthosteric site, yet remaining focused on the blockade of CCR5, allosteric antagonists have been developed for members of the GPCR superfamily that engage the receptor from its intracellular side, directly preventing G protein coupling, or even laterally from within the cell membrane, freezing conformational rearrangements leading to accommodation of G proteins (32, 33). GPCR homo and hetero dimerization, as well as oligomerization, extends the complexity of pharmacological intervention (34).

Overall, several possibilities lie ahead in the future of CCR5 therapeutics and the present knowledge of the structural details of this important receptor should provide the platform for their development.

## Conclusion

In conclusion, the elucidation of the fine structural details of the CCL5:CCR5 interaction, both in agonist and antagonist mode, allows the engineering of core-modified chemokine ligands that may surpass the available CCL5 derivatives in their potency and therapeutic concept. Moreover, new classes of CCR5 blockers may arise from the available 3D structural information and understanding of GPCR complexity.

## Data Availability Statement

The original contributions presented in the study are included in the article/supplementary material. Further inquiries can be directed to the corresponding author.

## Author Contributions

LV conceptualized and wrote the original draft of the manuscript. YA and LV prepared the models and figures. LV and YA reviewed and edited the manuscript. All authors contributed to the article and approved the submitted version.

## Funding

The research was funded by Nazarbayev University Grant to LV “Expanding the therapeutic landscape of CCR5 blockade and CCL5 engineering” (Code: 021220FD2551).

## Conflict of Interest

The authors declare that the research was conducted in the absence of any commercial or financial relationships that could be construed as a potential conflict of interest.

## Publisher’s Note

All claims expressed in this article are solely those of the authors and do not necessarily represent those of their affiliated organizations, or those of the publisher, the editors and the reviewers. Any product that may be evaluated in this article, or claim that may be made by its manufacturer, is not guaranteed or endorsed by the publisher.
